# Sagittal Spinal Alignment in Children and Adolescents: Associations with Age, Weight Status, and Sports Participation

**DOI:** 10.3390/children12050659

**Published:** 2025-05-21

**Authors:** Giada Annarumma, Fiore Mazza, Alessandro Ambrosi, Erica Keeling, Fredrick Fernando, Felice Sirico, Rossana Gnasso, Andrea Demeco, Marco Vecchiato, Maria Letizia Motti, Alessandro Biffi, Stefano Palermi

**Affiliations:** 1 Med-Ex, Medicine & Exercise, Medical Partner Scuderia Ferrari, 00187 Rome, Italy; giada.annarumma@gmail.com (G.A.); fioremazza@hotmail.it (F.M.); ale.ambrosi91@gmail.com (A.A.); ericakeeling47@hotmail.com (E.K.); fred.fernando@med-ex.it (F.F.); a.biffi@libero.it (A.B.); 2 Public Health Department, University of Naples Federico II, 80138 Naples, Italy; sirico.felice@gmail.com (F.S.); rossanagns@yahoo.it (R.G.); 3 Physical and Rehabilitative Medicine, Department of Medical and Surgical Sciences, University of Catanzaro “Magna Graecia”, 88100 Catanzaro, Italy; andrea.demeco@unicz.it; 4 Sports and Exercise Medicine Division, Department of Medicine, University of Padova, 35122 Padova, Italy; marcovecchiato.md@gmail.com (M.V.); letizia.motti@uniparthenope.it (M.L.M.); 5 Departmental Faculty of Medicine and Surgery, UniCamillus-Saint Camillus International University of Health Sciences, 00187 Rome, Italy

**Keywords:** sagittal index, pediatric posture, sports participation, pediatric health, BMI, children, adolescents

## Abstract

**Background**. Poor posture is a common musculoskeletal concern in children and adolescents and may lead to spinal discomfort and long-term structural issues. While excess weight has been linked to altered sagittal alignment, the impact of sports participation on spinal posture remains unclear. This study aimed to investigate the associations between weight status, sports participation, and sagittal spinal alignment in a pediatric population. **Methods.** This cross-sectional study was conducted within the “Ferrari Formula Benessere” corporate wellness program and included 698 children aged 5 to 16 years. Sagittal Index (SI) was measured using a standardized plumb line technique. Body mass index (BMI) was calculated and classified according to the WHO growth standards. Sports participation was self-reported and grouped into five categories: sedentary, skill-based, power-based, mixed, and endurance sports. **Results**. Age was the only significant independent predictor of the SI (β = 2.45, *p* < 0.001), with older children exhibiting higher SI values. Although a weak correlation was observed between BMI and SI (Spearman’s r = 0.24, *p* < 0.001), BMI was not a significant predictor when controlling for age. No significant differences in the SI were found between active and non-active children. Among sport disciplines, the SI was lowest in power-based sports (56.7 ± 22.3 mm) and higher in endurance (62.7 ± 24.4 mm), mixed (64.5 ± 23.2 mm), skill-based (61.1 ± 22.0 mm), and non-sport (64.2 ± 24.0 mm) groups, although these differences did not reach statistical significance (ANOVA *p* = 0.224). **Conclusions**. Age appears to be the primary factor associated with sagittal spinal alignment in children, while BMI and general sports participation showed no independent effect. Although some differences emerged between sport types, these findings were not statistically significant and should be interpreted with caution. These findings underscore the need for sport-specific, longitudinal research using objective posture assessment methods.

## 1. Introduction

One of the most common health problems in children and adolescents is poor posture [1]. It is often overlooked since it does not immediately interfere with daily activities and functioning. However, over time, poor posture can progressively deteriorate, potentially leading to musculoskeletal pain, compensatory movement patterns, and long-term spinal disorders [2,3,4]. Given the high prevalence of postural issues, it is crucial to understand the factors contributing to different types of postural misalignments and the conditions that exacerbate them [5].

The relationship between weight status and spinal alignment is complex and multifactorial. Several factors contribute to alterations in spinal curvature and sagittal alignment in overweight youth [6]. Overweight and obesity have been associated with increased lumbar lordosis and anterior pelvic tilt, which may affect overall sagittal balance [7]. Additionally, overweight children and adolescents often engage in more sedentary behaviors, such as prolonged sitting and excessive screen time, which may further contribute to postural misalignment [8]. However, not all overweight or obese children develop postural abnormalities, and other factors—including genetics, motor control, and physical fitness—likely moderate this relationship [9].

Regular physical activity, particularly in the form of structured sports participation, is widely recognized as a contributor to musculoskeletal development and postural health in children and adolescents [10]. Different sports place distinct biomechanical demands on the body, which may influence spinal alignment in various ways [11]. For example, disciplines such as gymnastics or dance often emphasize flexibility and core control. At the same time, sports involving high-impact or asymmetric loading—like weightlifting or tennis—may lead to different postural adaptations [12]. However, current evidence regarding how specific types of sports affect sagittal spinal alignment in pediatric populations remains limited and inconclusive.

Therefore, the present study investigated the associations between sagittal spinal alignment, weight status, and sport participation type in a large sample of children and adolescents. Specifically, we hypothesized that (1) older age and higher BMI would be associated with increased Sagittal Index (SI), reflecting the more anterior displacement of trunk posture, and (2) participation in certain sports, particularly power-based disciplines, would be associated with more favorable sagittal alignment compared to sedentary peers. Given the lack of longitudinal or interventional studies in this area, this exploratory, cross-sectional analysis was designed to identify associations and generate hypotheses for future research.

## 2. Materials and Methods

### 2.1. Study Design and Setting

The present study was conducted as part of the “Ferrari Formula Benessere”, a corporate wellness program managed by the Med-Ex, Medicine & Exercise, society, focused on primary health prevention [13]. This program occurs annually at the Ferrari company in Maranello (Mo), Emilia Romagna, Italy. As part of the initiative, a designated period is dedicated to the medical evaluation of employees’ children, who undergo various medical assessments conducted by specialists in different disciplines, including sports medicine, cardiology, pediatrics, orthopedics, dermatology, and nutrition [8,13]. Participation in the program is voluntary and free of charge for both employees and their families.

### 2.2. Study Population

The inclusion criteria for this study were children aged 5 to 16 years attending the Med-Ex sports medicine screening visit. Exclusion criteria included any history of musculoskeletal, neurological, or orthopedic disorders. Additionally, children classified as underweight (BMI < 5th percentile, *n* = 18; 2.5% of the total evaluated sample) were excluded from the final analysis. This subgroup was numerically limited, and existing evidence suggests that low body mass may independently affect spinal posture due to reduced muscle mass, bone mineral content, and altered postural control [14]. Including underweight children could therefore introduce bias and reduce the interpretability of findings related to BMI, sports participation, and sagittal alignment.

Over twelve weeks between October and December 2024, all eligible children were invited to participate in this study. There were no reported refusals or dropouts during recruitment. The assessments were conducted during the routine clinical visit performed by board-certified sports medicine physicians with expertise in pediatric musculoskeletal evaluation [12]. Four physicians participated in data collection across the study period. To minimize inter-observer variability in key measurements—especially those related to the Sagittal Index (SI)—a standardized training session was conducted before data collection. Physicians followed a shared protocol for anatomical landmark identification and measurement procedures. However, formal inter-rater reliability testing (e.g., intraclass correlation coefficient) was not performed, which remains a study limitation.

If eligible, children and their parents were approached for informed consent. Before participation, each child (or their parent/guardian) provided written informed consent, allowing for medical evaluation and data collection by Med-Ex. All data were recorded anonymously, and participants’ privacy was safeguarded according to ethical standards. This study adhered to the principles of the Declaration of Helsinki and its later amendments. Data were collected per standard privacy regulations, ensuring confidentiality and compliance with ethical guidelines. The local ethical committee approved this study.

### 2.3. Measurements

A. Nutritional Assessment

Children’s weight and height were assessed [8]. The measurements were taken using a TANITA MC-780MA electronic scale for weight and a GIMA Astra altimeter for height, following standardized procedures established in previous studies [8]. Weight was recorded to the nearest 0.1 kg and height was measured to the nearest 0.1 cm. BMI was calculated as weight (kg) divided by height squared (m^2^). Body composition (including percentage of body fat) was determined using bioelectrical impedance analysis (BIA) via the Tanita Analyzer [8].

Due to the many daily assessments, measurements were not performed on an empty stomach, and fluid and food intake were not controlled. The mean value of three consecutive weight and height measurements was used for the analysis to minimize variability.

Each child’s BMI percentile was classified using the WHO AnthroPlus software (version 1.0.1), categorizing participants as follows [15]:

- Underweight: Below the 5th percentile;
- Normal weight: 5th–84.9th percentile;
- Overweight: 85th–94.9th percentile;
- Obese: Above the 95th percentile.

B. Spine Evaluation

The SI is a clinical parameter used to assess global trunk sagittal balance. It reflects global sagittal trunk alignment by quantifying the horizontal displacement between the C7 and S2 spinous processes. A higher SI indicates greater posterior displacement of the upper trunk, which may reflect increased thoracic kyphosis or forward head posture. Age-specific thresholds are used to identify children at risk of hyperkyphosis [16].

In this study, the SI was measured following a procedure already described in the literature [16]:

1. The physician identified the C7 spinous process by palpating the cervical spine in a neutral position.
2. Localization of C7 was confirmed using the flexion–extension test.
3. The S2 spinous process was identified as the midpoint between the left and right posterior superior iliac spines (PSISs).
4. The plumb line technique was used:
  o A plumb line was stabilized against the patient’s parietal bone, ensuring alignment with the PSIS midpoint.
  o The distance between the plumb line and the reference points was measured using a rigid ruler.
  o The final SI was obtained by subtracting the S2 plumb line distance (PD) from the C7 PD.
5. A positive SI value indicated a posterior displacement of C7 relative to S2, while a negative value indicated an anterior displacement.

Based on previous research [16], the SI cut-offs were defined as follows:

- Children aged ≥ 9 years: SI > 95 mm (risk of hyperkyphosis);
- Children aged < 9 years: SI > 65 mm (risk of hyperkyphosis).

Other postural deformities (e.g., scoliosis, hyperlordosis) were not systematically assessed or quantified, as this research focused on exploring sagittal plane alignment and its associations with weight status and sports participation.

C. Sports Participation

Each child was asked about their sports activity during the evaluation, using validated questionnaires [17]. The type of sport was recorded and categorized into five main groups [12]:

0 No sport (sedentary);
1 Skill-based sports (e.g., gymnastics, dance);
2 Power sports (e.g., weightlifting, wrestling);
3 Mixed sports (e.g., soccer, basketball);
4 Endurance sports (e.g., long-distance running, swimming).

Since sports participation was self-reported during the clinical visit, no data were available on the training frequency, intensity, or duration. Recreational and competitive levels were not distinguished.

### 2.4. Statistical Analysis

Statistical analyses were performed using IBM SPSS Statistics for Windows, Version 26.0 (IBM Corp., Armonk, NY, USA) and Python 3.10 with the SciPy and Stats models libraries for effect size calculations. Continuous variables were expressed as means ± standard deviations (SDs), and categorical variables were reported as absolute values (*n*) and percentages (%). The Shapiro–Wilk test was used to assess data normality, and the chi-squared test was applied for categorical variable comparisons. Outliers were identified as values exceeding ±1.5 standard deviations from the mean SI and were excluded from subgroup analyses to reduce the influence of extreme values on group-level estimates and improve data reliability

Independent *t*-tests or Mann–Whitney U tests were used to compare two groups, depending on data distribution. One-way ANOVA or the Kruskal–Wallis test was applied for multiple group comparisons. Effect sizes were reported alongside *p*-values to enhance interpretability. Cohen’s d was calculated for pairwise comparisons, and Eta squared (η^2^) was reported for the ANOVA tests. Spearman’s rank correlation was used to explore associations between the SI and other continuous variables (e.g., BMI and age). A *p*-value < 0.05 was considered statistically significant.

## 3. Results

The final study sample included 698 children, with a balanced gender distribution (50% females) and a mean age of 9.42 ± 2.84 years. Table 1 presents the main anthropometric characteristics. The average BMI was 18.55 ± 3.93 kg/m^2^, and the mean BMI percentile was 66.17 ± 28.80. The mean Sagittal Index (SI) was 60.34 ± 20.59 mm. Based on established age-specific cut-offs, 34 children (4.87%) were classified as having a pathological SI.

**Figure 1:**
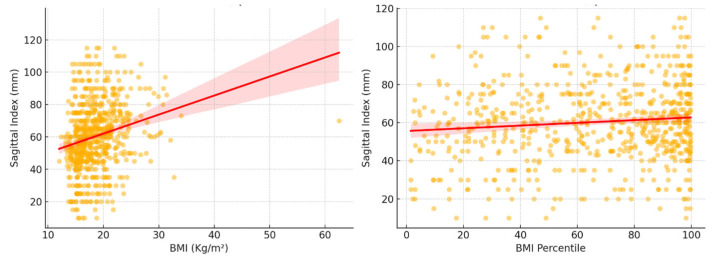
Correlation of Sagittal Index and weight measurements. On the left, the correlation between BMI and the SI shows a weak but statistically significant positive association. On the right, the correlation between BMI percentiles and the SI shows a weaker yet still substantial relationship.

A weak but significant positive correlation was observed between BMI and Sagittal Index (Spearman’s r = 0.24, *p* < 0.001), indicating that children with higher BMI tended to exhibit slightly increased SI values. A similar but weaker association was noted between BMI percentiles and SI (Spearman’s r = 0.08, *p* = 0.030). Figure 1 visually represents this association.

The analysis of SI values by sex, BMI classification, and sports participation revealed no significant differences (Table 2). Males had a mean SI of 61.0 ± 21.0 mm and females 59.7 ± 20.2 mm (*p* = 0.398, Cohen’s d = 0.06). Children with normal weight showed a mean SI of 59.9 ± 21.0 mm, while those classified as overweight or obese had 61.2 ± 19.8 mm (*p* = 0.407, Cohen’s d = 0.06). SI values were also similar between active (60.3 ± 20.8 mm) and non-active children (60.8 ± 19.2 mm) (*p* = 0.794, Cohen’s d = 0.02).

**Figure 2:**
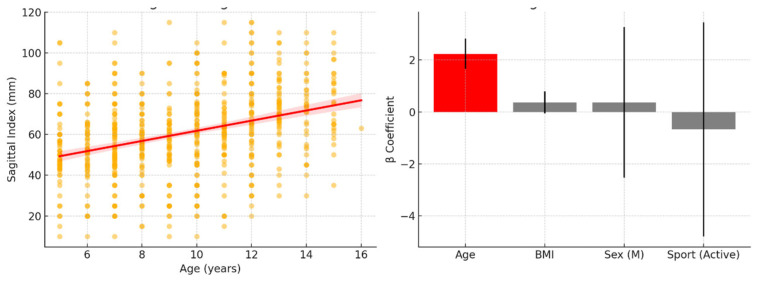
Regression analysis findings. On the left is a scatter plot illustrating the relationship between age and SI, with a clear positive trend line. On the right is a bar plot of regression coefficients (β values) for age, BMI, sex, and sports participation, with confidence intervals. Only age emerged as a significant predictor (highlighted in red), while all other variables showed non-significant contributions to SI.

The updated multiple regression analysis confirmed that age was the only significant independent predictor of the SI (β = 2.45, *p* < 0.001), indicating that the SI increases with age regardless of other variables (Figure 2). In contrast, BMI (*p* = 0.326), sex (*p* = 0.265), and sports participation (*p* = 0.760) were not statistically significant predictors. Although age was a statistically significant predictor, the model explained only 8.6% of the variance in the SI, indicating that most variability in sagittal alignment remains unexplained by the variables included in this analysis.

**Figure 3:**
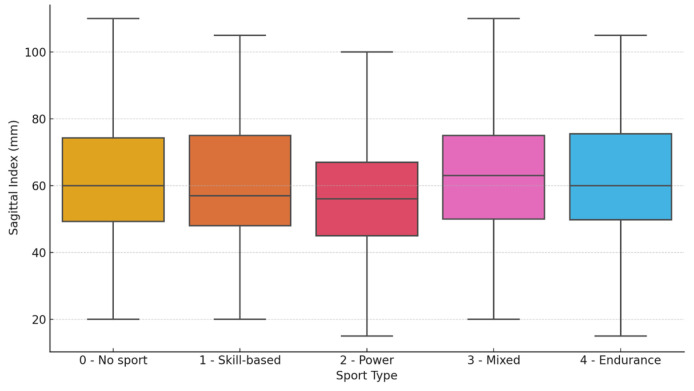
Differences in Sagittal Index across sport types: 0—no sport (sedentary), 1—skill-based sports, 2—power sports, 3—mixed sports, and 4—endurance sports.

The sedentary group (Category 0) included 103 children (58 girls, 45 boys—SI 64.2 ± 24.0 mm). The skill-based sports group (Category 1) consisted of 58 children (42 girls, 16 boys—SI 61.1 ± 22.0 mm), while the power-based sports group (Category 2) included 73 children (42 girls, 31 boys—SI 56.7 ± 22.3 mm). The mixed sports group (Category 3) was the largest, with 341 children (139 girls, 202 boys—SI 64.5 ± 23.2 mm), followed by the endurance sports group (Category 4—SI 62.7 ± 24.4 mm), which comprised 123 children (66 girls, 57 boys). A one-way ANOVA test showed no statistically significant differences in SI values across the five sport categories (F(4, 693) = 1.43, *p* = 0.224, η^2^ = 0.007) (Figure 3).

## 4. Discussion

This study investigated the relationship between weight status, sports participation, and sagittal spinal alignment in a large pediatric sample. The primary finding was that age is the strongest and only significant independent predictor of the SI, with older children showing higher SI values. Although a weak positive correlation was observed between BMI and the SI, BMI was not a significant predictor when controlling for age. Similarly, general sports participation did not show any significant association with the SI. These results suggest that age-related spinal growth and development may dominate sagittal alignment more than weight status or general physical activity.

Although we hypothesized that higher BMI would be associated with postural misalignment, BMI and BMI percentiles were not independent predictors of the SI in the multivariate model. This challenges previous research suggesting that excess weight contributes to spinal misalignment through increased mechanical load or compensatory postural adaptations [18,19]. However, differences in age ranges, postural measurement tools, and definitions of spinal misalignment may explain inconsistencies across studies. For instance, several of the studies reporting an association between BMI and spinal curvature used digital photogrammetry or radiographic analysis, which may detect subtler postural deviations than our plumb line method [20].

Concerning sports participation, SI values were not significantly different between active and inactive children, and the regression model confirmed that participation in sport (as a general variable) was not a significant predictor of the SI. Although children practicing power-based sports exhibited lower mean SI values, this observation was not statistically significant (ANOVA *p* = 0.224, η^2^ = 0.007) and may reflect the lower average age in that group rather than an actual sport-specific effect. Moreover, given the small effect size and lack of statistical support, we urge caution in interpreting this trend. Differences in training frequency, intensity, and type (recreational vs. competitive) were not captured, limiting the specificity of sport-related conclusions.

Notably, the SI values did not differ significantly by sex, and while males had a slightly higher SI than females, the effect size was negligible (Cohen’s d = 0.06). These findings suggest that sagittal alignment develops similarly across genders in this age group, at least within the measurement limits of this study. However, we did not explore whether SI trajectories differ across puberty stages, and future studies should stratify analyses by both age and pubertal development.

Our findings are consistent with prior studies indicating that age-related changes in spinal curvature are a key determinant of sagittal balance in childhood and adolescence [21,22]. During growth, natural increases in thoracic kyphosis and lumbar lordosis can alter the sagittal profile of the spine, with vertebral morphology and neuromuscular development playing central roles [23,24]. In this context, the limited influence of BMI or sports type observed in our analysis may reflect the predominant role played by developmental processes during childhood. The explanatory power of our regression model was modest (R^2^ = 0.086), indicating that most SI variability remains unexplained by age, BMI, sex, or sports participation. This suggests that other important contributors, such as neuromuscular control, muscle strength, flexibility, screen time, posture during sitting, and genetic predisposition, may substantially shape spinal alignment during growth [25]. These unmeasured variables should be incorporated in future models to improve predictive accuracy. While some adult studies report that resistance or strength-based training may improve spinal posture and reduce thoracic kyphosis [26,27,28,29], such effects may not be directly translatable to younger populations or detectable using clinical measurements such as the SI.

Several limitations should be acknowledged. First, this was a cross-sectional study, and causality cannot be inferred. Second, the SI was assessed using a plumb line and ruler, a manual method susceptible to inter-rater variability and measurement error. Although trained sports medicine physicians performed assessments, inter-observer reliability was not formally tested, and digital tools or 3D posture analysis systems would have provided greater objectivity. Third, sports participation was self-reported, and we lacked detailed information about training volume, frequency, and competitive level. Furthermore, although outliers were excluded (defined as values > ±1.5 SD from the mean), this process should be interpreted cautiously as it may affect reproducibility. Fourth, while we excluded underweight children due to their small number (*n* = 18), the potential impact of low BMI on spinal posture was not explored. Lastly, age distribution within gender groups was not analyzed, possibly introducing residual confounding.

These findings carry several practical considerations. First, clinicians and educators should recognize that age-related developmental changes primarily influence sagittal spinal alignment in children and adolescents. Variations observed during adolescence may reflect normal physiological growth rather than pathological postural deviations. Second, while strength-based sports were associated with lower SI values, this observation was not statistically significant and may have been influenced by age differences across sport groups. Therefore, any integration of strength-focused activities into pediatric training programs should be based on broader functional benefits, such as core stability and musculoskeletal health, rather than specific assumptions about postural correction. The lack of a clear association between general physical activity and SI underscores the need for more nuanced, sport-specific investigations rather than one-size-fits-all recommendations. Furthermore, the measurement of sports participation relied on self-reported data without details on duration, frequency, or intensity. This limits the ability to assess the true dose–response relationship between physical activity and spinal alignment. While our findings offer preliminary insights, the non-significant trends reported, particularly regarding power-based sports, should not be interpreted as evidence of causality. These associations require confirmation through well-controlled longitudinal or interventional studies. Additionally, although pathological SI cases were identified using established cut-offs, the small size of this subgroup (*n* = 34) precluded separate statistical analysis.

Future studies should explore longitudinal associations between sport-specific training and spinal development across age ranges. Intervention studies comparing modalities (e.g., strength-based vs. endurance-based exercise) with objective postural outcomes could help identify the most effective approaches to support spinal health. It will also be essential to include core strength, flexibility, motor control, and growth-related variables in future models to better understand the determinants of sagittal balance. Clearer outcome targets—whether reduction in the SI, improved postural control, or prevention of clinical dysfunction—should also be defined to inform intervention design.

## 5. Conclusions

This study highlights the central role played by age in determining sagittal spinal alignment in children and adolescents, with older participants exhibiting higher Sagittal Index values. While general sports participation and BMI were not independently associated with the SI, a non-significant trend toward a lower SI in children practicing power-based sports warrants further investigation. Given the limitations of cross-sectional design and manual measurement methods, these findings should be interpreted cautiously. Future research using objective postural assessment tools and longitudinal data is needed to clarify whether specific types of physical activity can positively influence spinal development. In the meantime, pediatric training programs may consider including strength-based activities as part of broader strategies to support musculoskeletal health during growth. Given the exploratory nature of this study, conclusions should be considered preliminary, and further investigation using objective tools and longitudinal designs is warranted.

## Figures and Tables

**Table 1:** Characteristics of sample size.

Age (years)	9.42 ± 2.84
Gender (females)	349 (50.0%)
Weight (Kg)	36.91 ± 15.45
Height (cm)	138.01 ± 17.28
BMI (Kg/cm^2^)	18.55 ± 3.93
BMI percentile	66.17 ± 28.80

Sagittal Index; BMI: body mass index.

**Table 2:** Comparison of Sagittal Index values between different categories.

Category	Group	SI	*p*-Value	Effect Size
Sex	Male	61.0 ± 21.0	0.398	Cohen’s d = 0.06
	Female	59.7 ± 20.2		
BMI	Normal weight	59.9 ± 21.0	0.407	Cohen’s d = 0.06
	Overweight + obese	61.2 ± 19.8		
Sport	Active	60.3 ± 20.8	0.794	Cohen’s d = 0.02
	Non-active	60.8 ± 19.2		

SI: Sagittal Index; BMI: body mass index.

## Data Availability

Data are available on reasonable request to the corresponding author due to privacy restriction.

## Abbreviations

The following abbreviations are used in this manuscript:

SI	Sagittal Index
BMI	Body mass index
